# Civil Liability of Regional Health Services: The Case of the Piedmont Region

**DOI:** 10.3390/ijerph18199954

**Published:** 2021-09-22

**Authors:** Alessandro Rizzi, Enrico Sorano, Stefano A. Cerrato, Federico Riganti, Alessandro Stiari, Ernesto Macrì, Alberto Sardi

**Affiliations:** 1 Department of Management, University of Turin, 10134 Turin, Italy; enrico.sorano@unito.it (E.S.); stefano.cerrato@unito.it (S.A.C.); alberto.sardi@unito.it (A.S.); 2 Department of Law, University of Turin, 10124 Turin, Italy; federico.riganti@unito.it; 3 A.O.U. Città della Salute e della Scienza di Torino, 10126 Turin, Italy; astiari@cittadellasalute.to.it; 4 Macrì Law Office, 00197 Rome, Italy; avv.emacri@gmail.com

**Keywords:** civil liability, patient safety, healthcare facilities, self-retention, insurance, national health service, regional health service, piedmont region

## Abstract

Civil liability represents one of the main responsibilities for healthcare facilities; it is the legal responsibility of paying money for damage to a person’s health. Even though this responsibility plays a key role in the economic sustainability of healthcare facilities, the literature does not enough investigate this responsibility in regional health services. The paper aims to define the evolution of compensation models for health civil liability adopted by regional health services. Through a longitudinal case study, the paper investigates the compensation model by a leading regional health service. The finding highlights the evolution of the compensation model for health civil liability adopted by a leading Regional Health Service from 1990 to 2021. It describes a transition from an insurance model to a mixed model based on self-coverage up to a set economic level, an insurance policy with self-insurance retention and deductible for all claims. The research contributes to the literature and practice throughout the definition of a compensation model for damages based on self-insurance of regional health service and insurance policies. The research promotes a compensation model used by a leading regional health service.

## 1. Introduction

Civil liability is the responsibility of the company for the damages involuntarily caused by the company’s activities and personnel in any capacity operating at the same structures. For instance, a healthcare facility undertakes to pay the damages caused to third parties for death, personal injury and property damage, as a result of medical harm occurring during its healthcare activities; a healthcare facility pays the damages if the recommendations were not respected [1,2,3]. The Institute for Healthcare Improvement defined medical harm as: “unintended physical injury resulting from or contributed to by medical care (including the absence of indicated medical treatment), that requires additional monitoring, treatment, or hospitalization, or that results in death” [4]. To avoid medical harm, healthcare facilities pay attention to patient safety [1,2,3], discipline emerged with the evolving complexity in healthcare systems and the resulting rise of patient harm in healthcare facilities [1,2,3].

The levels of avoidable harm in healthcare around the world remain unacceptably high [2], although the great attention on patient safety given by healthcare facilities. As highlighted by the World Health Organization recently [5], adverse events are one of the main reasons for disability and death in the world [6]. Slawomirski et al. demonstrated that 1 in 10 patients is harmed while receiving hospital care in industrialized countries [7]. Globally, as many as 4 in every 10 patients are harmed in primary and outpatient healthcare [5].

Despite the healthcare facilities are increasing the adoption of patient safety systems, 15 percent of total hospital activity and expenditure is still a direct result of adverse events in the OECD countries [7]. The healthcare systems have human and economic costs avoidable or preventable [2], beyond 80 percent of harm is preventable [8]. For instance, 50 percent of adverse events being preventable [9]. An example of prevention is engaging patients, which can reduce the harm by up to 15 percentage [8]. Most errors are related to diagnosis, prescription and the use of medicines [9].

Even though a strong investment in reducing patient harm can lead to significant financial savings [7], the risk is not disposable [3]. To cover this disposable risk, civil liability represents one of the main guarantees for patients; it gives to patients the right to compensation for damage due to medical errors, but the increase of compensation claims hard testing the economic sustainability of healthcare services [10]. Notwithstanding that it plays a key role in economic sustainability, literature does not enough investigate the civil responsibility in healthcare facilities [1,11,12]. The great challenge is the definition of an effective compensation model for health civil liability to limit the financial damage and guarantee the compensation of damages to patients [1]. This challenge is greater in regional health services because they have difficulty in taking out adequate insurance policies for the high premiums and deductibles applied by insurance companies [13].

To answer this challenge, the paper aims to define the evolution of the compensation model for health civil liability adopted by regional health services. It answers the following research questions:

How is the compensation model for health civil liability evolving in a leading regional health service?

**Figure 1:**
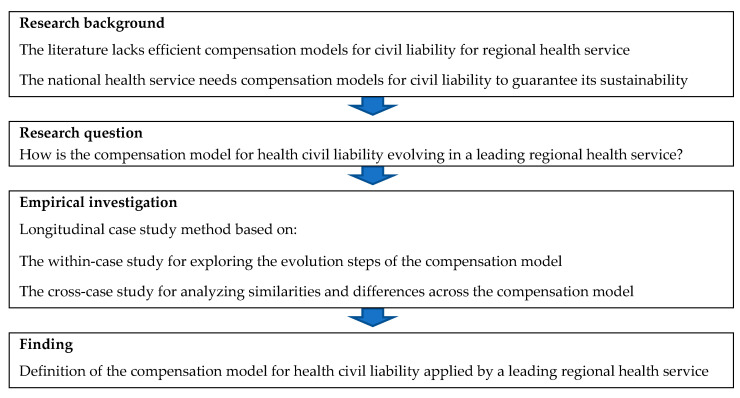
A synthesis of the research process.

To answer this research question, the authors carried out the research process synthesized in Figure 1. Firstly, it highlights the research background; it is focused on gaps of literature and practice. Secondly, it underlines the research question. Thirdly, it identifies the methodology approach adopted for maximizing the research validity. Fourthly, it describes the evolution of the compensation model used for damage applied in a leading regional health service. The result describes the compensation models for health civil liability adopted by Piedmont Health Service; it highlights a transition from an insurance model to a mixed model.

The next sections describe the research background with a specific focus on different models of compensation for damages applied in regional health services and the method adopted for the study. The finding section introduces the compensation model for civil liability adopted by Piedmont Health Service. The conclusion section summarizes the contributions, limitations and future opportunities.

## 2. Research Background

The patient safety discipline aims to prevent and reduce risks, errors and harm that occur to patients during healthcare; it is based on continuous improvement based on learning from errors and adverse events [5]. It includes a set of activities that creates cultures, processes, procedures, behaviors, technologies and environments in healthcare that consistently and sustainably lower risks, reduce the occurrence of an adverse event, avoidable harm, make error less likely and reduce its impact when it does occur [3], Brennan et al. defined adverse event as “injuries caused by medical management, and of the subgroup of such injuries that resulted from negligent or substandard care” [13], whilst Jacobs “an adverse event is a complication that is associated with a healthcare intervention and is associated with suboptimal outcome” [14]. Adverse events can be preventable or non-preventable [15]. An adverse event attributable to error is a preventable adverse event [13].

To contain the errors, healthcare organizations have introduced processes to manage clinical risk and guarantee patient safety [3,16]; these processes aim to analyze and prevent risks, also through the involvement of health personnel [11,17]. Some techniques applied are Incident Reporting, Sentinel Events, Root Cause Analysis, Failure Mode and Effect Analysis and Cartorisk. Incident Reporting is a system through which the operators report an adverse event to the risk manager, i.e., an event that has deviated from what was expected, through the use of anonymous cards [18,19]. Sentinel Events technique consists of reporting a particularly dangerous event that can have important consequences on the patient, but which can be avoided [20]. Root Cause Analysis recognizes the factors that lead to an adverse event causing weak performance [21,22]. Failure Mode and Effect Analysis identifies and eliminates the causes of an error before it occurs and it is a widely used method in healthcare as it allows to have high reliability of the values analyzed even in large organizational contexts [23,24,25]. Cartorisk analyzes the main clinical processes for the healthcare services aimed at identifying and assessing risks in advance [1,11].

Despite the adoption of these techniques, the risk is not disposable [2]. Risk is defined as a potential condition or event, intrinsic or extrinsic to the process, which can change the expected outcome of the process [26]. It is measured as the product between the probability of a specific event occurring and the severity of the resulting damage; the ability of the human factor to identify in advance and contain the consequences of the potentially harmful event limits the risk [27]. In the healthcare sector, clinical risk is the probability that an individual is the victim of an adverse event, that is, suffers damage or discomfort attributable, even if involuntarily, to medical care provided during the hospitalization, which causes an extension of the hospitalization, worsening of health conditions or death [27,28]. 

To cover the damages caused to patients, healthcare facilities must adopt an efficient model of compensation. The adoption of an efficient model plays a key role in the economic sustainability of healthcare facilities; optimal civil liability management also represents a guarantee for patients who have suffered the damage, ensuring compensation even if the healthcare facilities that caused it does not have sufficient assets on which to claim [10]. The compensation models adopted by healthcare facilities for damage concerning their activities are three: insurance, direct and mixed [29]. 

The insurance model includes the stipulation of an insurance policy to cover the health civil liability in which there is insurance coverage without or with a minimal deductible. The direct model, called self-retention or self-insurance, does not include any insurance policy and the entire risk is in charge of the health authority; it is recognized as an investment, but it does not reduce the probability and severity of a financial loss. The mixed model is a set of the two models described above in which there is an insurance policy for the coverage of civil liability with a deductible and/or Self-Insurance Retention—SIR, i.e., the amount established for each claim that the insured person covers with his resources [30]. 

To highlight the models adopted by the OECD country that has best contained the increase of healthcare spending in the last 10 years, i.e., Italy (https://stats.oecd.org/ access on 10 August 2021), the paper illustrates the application of three compensation models for damages adopted by the Italian Regions (see Table 1) (https://www.agenas.gov.it/ access on 10 August 2021):

➢ insurance model = insurance companies cover the risk and any damage
➢ direct model = health authorities self-cover the risk and any damage
➢ mixed-model = a mix of the two models described above.

The Italian Regions have applied different compensation models for civil liability [29]. To know the model choice of a leading regional health service, the paper highlights a longitudinal case study on Piedmont Health Service as considerate the best regions according to a quality and efficiency index.

## 3. Method

The paper adopted a longitudinal case study methodology; it is an empirical investigation for a better understanding of a real problem [31]. This methodology has been adopted in numerous research to favor the exploration of complex situations allowing the researchers to describe a phenomenon within its context [32,33]. It is based on several observations of the phenomenon over a period [31]. The research was developed through three different stages: case study selection, data collection, data analysis.

Case study selection. To select a leading regional health service, the research adopted the index suggested by the Italian Ministry of Health, i.e., Index Quality and Efficiency (IQE). This index includes the evaluation of 19 parameters:

1. Score of the Essential Levels of Assistance Grid
2. % incidence of surplus/deficit on ordinary operations
3. Average pre-operative hospital days
4. % of operations for fracture of the femur operated within two days
5. % discharged from surgical wards with medical Diagnosis Related Groups (DRGs)
6. % of hospitalizations with surgical DRGs out of total hospitalizations
7. % of ordinary hospitalizations with DRGs at high risk of inappropriateness
8. % of diagnostic daytime hospitalizations out of total daytime hospitalizations with medical DRGs
9. % of medical cases with hospitalization beyond the threshold for patients with age ≥ 65 years out of the total medical hospitalizations with age ≥ 65 years
10. Deviation from the standard envisaged for the incidence of expenditure for collective assistance on the total expenditure (5%)
11. Deviation from the expected standard for the incidence of district assistance expenditure on the total expenditure (51%)
12. Deviation from the expected standard for the incidence of hospital care expenditure on the total expenditure (44%)
13. Expenditure per capita for basic healthcare
14. Expenditure per capita for drugs
15. Average cost of hospitalization for acute cases in ordinary hospitalization
16. Average cost for post-acute hospitalization
17. Expenditure for clinical activity
18. Expenditure for laboratory
19. Expenditure for diagnostics.

According to the IQE, the research investigates the model applied by the Piedmont Region—Italy. It has been selected due to it is recognized as the first in the ranking among the benchmark Regions for National Health Service—Deliberation of Italian State-Regions Conference No. 21/2019. The ranking is drawn up by the Commission of the Italian Ministry of Health, in charge of evaluating the best regional performances and selecting the benchmark Regions to determine the distribution of the national health fund. The Piedmont Health Service has an IQE of 10, which was the highest among the Italian Regions. 

Data collection. This stage allows researchers to systematically collect data on research objects and on the settings in which they occur [31]. Data was collected through the study of legislation and official documents, semi-structured interviews and direct observation [31]. This data was collected by four researchers in specific Excel sheets from January 2020 to December 2020.

Firstly, the researchers collected data on (a) National and Regional regulations, (b) Piedmont Region—Population (inhabitants, province, land area, density) and Piedmont Heath Service (healthcare expenditure, employees, local health authorities, university hospitals authorities, hospitals, healthcare beds), and (c) compensation model for health civil liability. The information was collected from the following websites.

✓ https://www.gazzettaufficiale.it/ *
✓ https://www.regione.piemonte.it/ *
✓ https://www.salute.gov.it/ *
✓ https://www.salutepiemonte.it/ *
✓ https://www.agenas.gov.it/ *
✓ https://www.nuovistrumenti.it *

* Access on 10 August 2021

Secondly, the researchers interviewed 6 risk and administrative managers of important regional healthcare facilities and 3 risk managers of insurance companies to confirm or integrate the document collection. Thirdly, the researchers observed the main managerial practices adopted by the Regional Health Service. The documents collected aimed to understand the maximum and deductible for claim, maximum for claims per year, paying entity and management entity.

Data analysis. After data collection, the researchers were analyzed through the within-case study and the cross-case study [31].

The within-case study is an in-depth exploration of a unique case as a single entity [34]. Through this analysis the researchers analyzed:

➢ The main National and Regional Rules to identify the main regulations of the Italian National Health Service and Piedmont Health Service
➢ The main information on Piedmont Region to identify the main characteristics of Piedmont Health Service
➢ The main evolution steps of the compensation model for health civil liability.

The cross-case study analyses similarities and differences across the compensation model adopted by Piedmont Health Service [35]. Through this analysis the researchers analyzed the different models according to the items suggested by the regulations of Piedmont Health Service:

➢ Maximum amount and deductible for a single claim
➢ Maximum amount for total claims per year
➢ Paying Entity
➢ Management entity of claims
➢ Special regional fund.

Data triangulation was achieved by using multiple sources of information [31,36,37]. Multiple resources supported the researchers to crosscheck data and verify the validity of the data obtained. Through case study methodology, the researchers used multiple pieces of evidence to improve the context understanding and, consequently, the research contribution [31].

## 4. Results

The within-case study analyses the regulations (Table 2), the main information on Piedmont Health Service (Table 3) and the evolution steps of the compensation model for health civil liability adopted by the Piedmont Health Service (Table 4).

Since 1978, the Italian National Health Service, subdivided into Regional Services, provides healthcare to those who need it without social, individual and economic differences, guaranteeing everyone the same treatment (principles of universality, equality and equity). In the last two decades, the National Health Service has had fewer financial resources, although it provides the same performance in terms of quality and safety. Due to poor financial resources, the Italian legislature has promoted initiatives to improve the efficiency of healthcare services. Two of the main initiatives are the Balduzzi Law No. 158/2012 and the Gelli-Bianco Law No. 24/2017. The Balduzzi Law 2012 introduced the responsibility of healthcare operators through two articles: art. 3 and art. 3-bis. The Gelli-Bianco Law 2017 regulated the safety of care and the assisted person; it also introduced compulsory insurance up for the public or private health or social-healthcare facilities, to cover third parties for damages (art. 10); however, this article still lacks implementing decrees. Article 7 of the Gelli-Bianco Law specifically regulates the civil liability of public and private healthcare facilities as well as of the sanitary operators. Furthermore, the legislator has introduced the insurance obligation also for doctors, as a consequence of the growth in requests for compensation for damages advanced by patients against doctors for the past decades [1].

The main features of the Piedmont Region are illustrated in Table 3. Piedmont Region holds 4.3 million inhabitants on 25,400 square kilometers. Piedmont Health Service has a turnover of €8.5 billion approximately, €2000 healthcare expenditure per capita, 55,000 employees, 12 Local Health Authorities and 11.472 healthcare beds. The regional health service of the Piedmont Region includes excellent healthcare facilities such as the University Hospital City of Health and Science of Turin. It is the largest healthcare center at the national and European level and has excelled in many disciplines such as Oncology, Pediatrics, Orthopedics, Gynecology, etc. (https://www.cittadellasalute.to.it/ access 15 July 2021). This healthcare center includes: (i) Molinette: the third largest hospital in Italy and the first in the complexity of care; (ii) Sant’Anna: hospital specialized in the treatment of female gender’s diseases; (iii) Regina Margherita: a qualified unit in the treatment of infants’ diseases and (iv) Orthopedic Trauma Center: regional trauma center, severe burns, neurosurgery, neurorehabilitation.

Below, the paper analyses the main evolution steps of the compensation model adopted by Piedmont Region Service. 

Until the 1990s, insurance companies managed the claims of the Piedmont Health Service through the signing of an insurance policy. The compensation claims were limited which allowed Piedmont Health Service to pay a minimum insurance premium.

In the second half of the 1990s, compensation claims have grown; furthermore, the judgment of the Courts has changed with sentences in favor of patients. This situation had seen insurance companies increase the compensation and, consequently, increase the premiums and decrease the guarantees to recover the costs. Due to situation, the Regional Health Service adopted self-claim management because the premiums became economically unsustainable for the Regional Health Authority. 

In the early 2000s, a great event changed the management of civil liability in the Piedmont Region, i.e., defective heart valves were installed. There were many claims for damages for this accident and the insurance company paid great compensation. 

After that, the Piedmont Region had difficulty stipulating insurance agreements for high premiums; accordingly, the Piedmont Region adopted a mixed model from 2005, considered the most suitable among the three models. The main actors of the mixed model are the Regional Health Authorities and the insurance companies. The adoption of the mixed model was dictated by the need to limit the expense for compensation damages. Piedmont Health Service has applied different typologies of mixed models during the time according to specific items (see Table 4). 

It is useful to underline that all mixed models adapted are developed on different items. One of the most items is the Special regional fund established by Regional Law No. 9/2004 (art. 21); it aims to pay the claims of the Regional Health Authorities for an amount between the minimum deductible and the maximum amount established at the signing of the insurance contract. All Health Authorities of the Piedmont Health Service finance the Special regional fund through a subdivision based on two criteria established by Deliberation of Piedmont Region Deliberazion No. 68/2019:

1. Average previous claims of the Health Authorities calculated on a 10-year of the claims paid (weight 80%).
2. Dimension healthcare facilities calculated based on the total salaries of healthcare personnel (weight 20%).

The calls of patients damaged for judgment had increased steadily from 2010 to today; it has led to the Piedmont Health Service to manage more compensation claims [38]. 

The main evolution steps of the compensation model for health civil liability are highlighted in Table 4. The cross-case study analyses similarities and differences across the mixed model adopted by Piedmont Health Service. Before analyzing similarities and differences, Table 5 represents the mixed model from 2005 to 2021.

**Figure 2:**
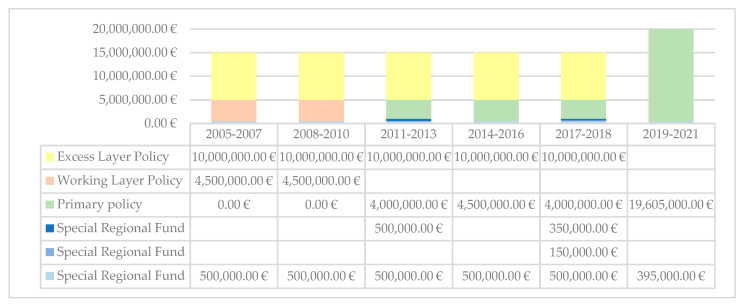
Maximum amount for a single claim.

As highlighted in Figure 2, the maximum amount for a single claim moved from €15 million (2005–2018) to €20 million (2019–2021). The first mixed model was based on 4 guarantees, i.e., Special regional found (Piedmont Health Service), and Primary Policy, Working Layer Policy and Excess Layer Policy (3 insurance companies). In the first model, the special regional found covered €500,000 with deductible of €1.500, Primary Policy Up to €500,000 only if Special regional fund is exhausted, Working Layer Policy covered 5 million with deductible of €500,000, and Excess Layer Policy covered 15 million with deductible of €5 million. The last mixed model adopted for 2019–2021 is based on 2 guarantees, i.e., Special regional found (Piedmont Health Service), and Primary Policy (insurance company). It introduces the SIR of 39% up from €395,000 to €500,000.

**Figure 3:**
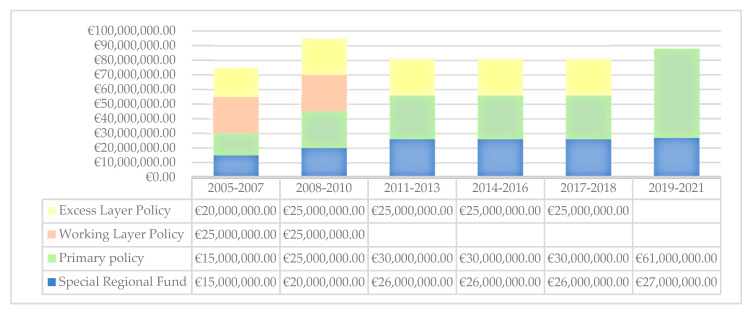
Maximum amount for total claims per year.

As highlighted in Figure 3, the maximum amount for annual claim moved from €75 million to €88 million. It describes a stable amount, however, what changes are the division of risks between the various insurance companies; the risk moves from three insurance companies to an insurance company.

Firstly, the last mixed model adopted from 2019 to 2021 presents a new formula; it includes the SIR. This formula is based on an amount for all claims of € 395,000 with a SIR of 39% paid by the Special regional fund. The introduction of a SIR allows the charge of an amount established in a health civil liability insurance policy that is paid by the insured immediately. Under a health civil liability policy written with self-insurance retention, the regional health service, rather than the insurance company, pay defense and/or expenditure associated with a claim until the self-insurance retention limit was reached. In contrast, under a policy written with a deductible provision, the insurer would pay the costs associated with a claim on the insured’s behalf and then seek reimbursement of the deductible payment from the insured. In opposite, under a policy written with deductible, the regional health service pays the costs associated with a claim on the insured’s behalf and consequently asks for a refund from the insurance company.

Secondly, the amount of the Special regional fund has had a steady increase in almost all renewals from €15 million (2005–2007) to reach €27 million (2019–2021). The most reason for the increase of the Special regional fund is the increasing number of claims in charge of the Regional Health Service, also as a response to the introduction of Balduzzi Law and Gelli-Bianco Law. The increase of Special regional fund commits important economic resources. On the one hand, it stops the financial resources of the National Health Service in the historical period of poor resources; on the other hand, it decreases the insurance premium while maintaining high guarantees.

In light of the above analysis, the research describes a trend toward claim management of catastrophic damage where the support of insurance companies goes in two directions. First of all, claim management needs great technical competencies, e.g., legal, economic, insurance. Health facilities rarely dispose of this skill, knowledge and abilities. In this case, insurance companies assist claim management through technical support. Secondly, this model gives economic safety capable of promoting the sustainability of the health service and the warranty to patients of compensation for damage due to medical errors.

## 5. Conclusions

The paper investigated the compensation model for damages concerning a leading regional health service. Through a longitudinal case study, it highlighted the evolution of the model adopted by a leading Regional Health Service, i.e., Piedmont Region. It describes a national context, i.e., Italy, where the Regional Health Authorities have difficulty in taking out insurance policies for the high premiums and deductibles applied by insurance companies because they have to pay high compensation to injured third parties [29]; the Italian Regions face this problem by adopting a heterogeneous behavior dictated by the search to find the best formula.

The findings of this paper highlight the evolution of compensation models for health civil liability applied to the Piedmont Region. It shows a transition from an insurance model (1990–2004) to a mixed model (2005–2021). This transition was decided as a consequence of events such as accidents and standards. The novelty of the findings consists of the description of a mixed model based on an insurance policy with a fixed deductible for all claims, self-retention coverage up to a set economic level, and a percentage of self-retention for Regional Health Authorities with insurance coverage part of the compensation. 

The paper contributes to the literature and practices through the definition of a compensation model for damages that promotes the economic sustainability of regional health services ensuring compensation to patient even if the healthcare facilities that caused it does not have sufficient assets on which to claim. Furthermore, it highlights the strategies of a leading Regional Health Authority to reduce the compensation for damage and guarantee the performance of the healthcare services.

The research implications give, on the one hand, the chance for scholars to analyze the model adopted by this Regional Health Service to improve and/or compare it with other regional systems; on the other hand, the implications give the chance to operators in the healthcare sector, such as managers, consultants, professionals, to know the model adopted by a leading regional health service. 

The main limitation of this research is the study of a unique regional health service. This limitation made it possible to deepen the single case study in detail and provide a better contribution. 

Future opportunities concern the possibility to improve and compare the results obtained with other regional authorities. This paper can represent a first working paper on this topic, however, this research area needs new research useful for improving the literature and practice to define the best formula for the compensation model for civil liability.

## Figures and Tables

**Table 1:** The models of compensation for damages.

Region	Insurance Model	Direct Model	Mixed Model
Abruzzo	X		X
Basilicata		X	
Calabria	X		
Campagna	X	X	
Emilia Romagna		X	
Friuli Venezia Giulia			X
Lazio	X	X	X
Liguria		X	
Lombardy	X		
Marche	X	X	
Molise	X		
Piedmont			X
Puglia	X	X	X
Sardinia	X	X	X
Sicily		X	
Tuscany		X	
Umbria			X
Valle d’Aosta	X		
Veneto			X

**Table 2:** Main regulations on National Health Service and Piedmont Heath Service.

Main Regulations	Description
Art. 32 of 1948 Constitution	Protect health as a right of the individual and a collective interest
Law No. 833 of 1978	Establish the National Health Service and guaranteed health protection
Law No. 421 of 1992	Reorganize the National Health Service by corporatization
Leg. Decree No. 229 of 1999	Define the criteria of appropriateness, economy and scientific evidence in the choices of resources
DPCM No. 29 of 2001	Define the Essential Levels of Assistance
Regional Law No. 9 of 2004	Introduce the Special Regional Found for claim settlement
Min. Decree of 2009	Establish Information System for Monitoring Errors in National Health Service
Law No. 15 of 2009	Introduce a performance measurement system to improve the productivity of public employees
Leg. Decree No. 158 of 2012	Introduce the monitoring of health risks to prevent litigation and reduce insurance charges
Min. Decree No. 70 of 2015	Define the qualitative, structural, technological and quantitative standards relating to health care
Stability Law 2016	Introduce the clinical risk management in the National Health Service
Law No. 24 of 2017	Introduce the patient safety, the assisted person and the responsibility of healthcare operators

**Table 3:** Main information on Population, and Piedmont Health Service.

**Piedmont Population 2021**	**Data Population**
Total	4.3 million
Province	No. 8 *
Land area	25,400 km^2^
Density	168/km^2^
**Regional Healthcare Service**	**Data regional Healthcare Service**
Turnover	€8.5 billion approximately
Healthcare expenditure	€2000 per capita
Employees	55,000 approximately
Local Health Authorities	No. 12
University Hospitals	No. 3
Hospitals Authorities	No. 48
Healthcare beds	No. 11.472

* Alessandria, Asti, Biella, Cuneo, Novara, Turin, Verbania-Cusio-Ossola and Vercelli.

**Table 4:** The main steps of the mixed model development.

Period	Steps of the Mixed Model Development
1990–2004	Piedmont Health Service adopted an insurance model
1990–2004	Patients increased the compensation claims and the insurance companies paid more settlements
1990–2004	Insurance companies increased premiums and reduced guarantees
2000–2002	Piedmont installed defective heart valves; this event caused an increase in compensation claims
2005–today	Piedmont adopted a mixed model; it included the creation of a Special regional fund
2005–today	Special regional fund increased steadily
2009–2015	Calls of patients damaged for judgment had increased steadily
2010–2019	Piedmont Health Service increased the number of claims managed

**Table 5:** Mixed models adopted by the Piedmont Health Service from 2005 to 2021.

Period	Max for Claim—Deductible for Claim	Max Claims/Year	Paying Entity	Management Entity
**2005–2007**	Up to €1500	Unlimited	Health Authority	Health Authority
	Up to €500,000—deductible €1500	€15 million *	Piedmont Health Service	Loss Adjuster
	Up to €500,000—deductible €1500 if Special regional fund is exhausted	€15 million	Insurance Company by Policy, namely Primary Policy	Insurance Company
	Up to €5 million—deductible €500,000 only for claims > € 500,000	€25 million	Insurance Company by Policy, namely Working Layer Policy	Insurance Company
	Up to €15 million—deductible €5 million only for catastrophic damage	€20 million	Insurance Company by Policy, namely Excess Layer Policy	Insurance Company
**2008–2010**	Up to €1500	Unlimited	Health Authority	Health Authority
	Up to €500,000—deductible €1500	€20 million *	Piedmont Health Service	Loss Adjuster Claim Committee
	Up to €500,000—deductible €1500 if Special regional fund is exhausted	€25 million	Insurance Company by Policy, namely Primary Policy	Insurance Company
	Up to €5 million—deductible €500,000 only for claims > €500,000	€25 million	Insurance Company by Policy, namely Working Layer Policy	Insurance Company
	Up to €15 million—deductible €5 million only for catastrophic damage	€25 million	Insurance Company by Policy, namely Excess Layer Policy	Insurance Company
**2011–2013**	Up to €5000	Unlimited	Health Authority	Health Authority
	Up to €1 million—deductible €5000 for childbirth and sentinel events claims Up to €500,000—deductible €5000 for different claims	€26 million *	Piedmont Health Service	Loss Adjuster Claim Committee
	Up to €5 million—deductible €1 million for childbirth and sentinel events claims Up to €5 million—deductible €500,000 for different claims	€30 million	Insurance Company by Policy, namely Primary Policy	Insurance Company
	Up to €15 million—deductible €5 million only for catastrophic damage	€25 million	Insurance Company by Policy, namely Excess Layer Policy	Insurance Company
**2014–2016**	Up to €5000	Unlimited	Health Authority	Health Authority
	Up to €1 million—deductible €5000 for childbirth and sentinel events claims Up to €500,000—deductible €5000 for different claims	€26 million *	Piedmont Health Service	Loss Adjuster until 30 June 2016
	Up to €5 million—deductible €1 million for childbirth and sentinel events claimUp to €5 million—deductible €500,000 € for different claims	€30 million	Insurance Company by Policy, namely Primary Policy	Insurance Company
	Up to €15 million—deductible €5 million only for catastrophic damage	€25 million	Insurance Company by Policy, namely Excess Layer Policy	Insurance Company
**2017–2018**	Up to €5000	Unlimited	Health Authority	Health Authority
	Up to €1 million—deductible €5000 for childbirth and sentinel events claims Up to €650,000—deductible €5000 for death claimsUp to €500,000—deductible €5000 for difference claims	€26 million *	Piedmont Health Service	Claim Committee Insurance Company
	Up to €5 million—deductible €1 million for childbirth and sentinel events claims Up to €5 million—deductible €650,000 for death claims Up to €5 million—deductible €500,000 for difference claims	€30 million	Insurance Company by Policy, namely Primary Policy	Claim Committee Insurance Company Health Authority
	€15 million deductible €5 million only for catastrophic damage	€25 million	Insurance Company by Policy, namely Excess Layer Policy	Insurance Company
**2019–2021**	Up to €5000	Unlimited	Health Authority	Health Authority
	Up to €395,000 deductible €5000 and SIR of 39% up to €500,000	€27 million *	Piedmont Health Service	Claim Committee Insurance Company
	Up to €20 million—deductible SIR of 39% up to €500,000	€61 million	Insurance Company by Policy, namely Primary Policy	Claim Committee Insurance Company Health Authority

* Special regional fund.
